# Analysis of Susceptibility to Selected Antibiotics in *Klebsiella pneumoniae, Escherichia coli, Enterococcus faecalis* and *Enterococcus faecium* Causing Urinary Tract Infections in Kidney Transplant Recipients over 8 Years: Single-Center Study

**DOI:** 10.3390/antibiotics9060284

**Published:** 2020-05-26

**Authors:** Olga Maria Rostkowska, Robert Kuthan, Anna Burban, Jagoda Salińska, Michał Ciebiera, Grażyna Młynarczyk, Magdalena Durlik

**Affiliations:** 1 Department of Transplantation Medicine, Nephrology, Internal Diseases, T. Orłowski Institute of Transplantation, Medical University of Warsaw, 59 Nowogrodzka Street, 02-006 Warsaw, Poland; ania.burban@gmail.com (A.B.); jagoda.salinska@gmail.com (J.S.); magdalena.durlik@wum.edu.pl (M.D.); 2 Chair and Department of Medical Microbiology, Medical University of Warsaw, 5 Chałubińskiego Street, 02-004 Warsaw, Poland; grazyna.mlynarczyk@wum.edu.pl; 3 Second Department of Obstetrics and Gynecology, Center of Postgraduate Medical Education, 80 Cegłowska Street, 01–809 Warsaw, Poland; michal.ciebiera@gmail.com

**Keywords:** urinary tract infections, kidney transplant recipient, antimicrobial resistance, drug resistance, infections, kidney, transplantation, *Klebsiella*, *Escherichia*, *Enterococcus*

## Abstract

**Background:** Urinary tract infections (UTIs) are the most common bacterial infections among kidney transplant (KTX) recipients. The purpose of this study was to analyze antimicrobial resistance (AMR) in four most common pathogens responsible for UTIs in KTX recipients and determine risk factors (RF) for resistance in the same group. **Methods:** Analyzed antibiograms were based on urine samples positive for bacterial growth of 10^5^ colony-forming units (CFU)/mL obtained from hospitalized adult KTX recipients presenting with UTI symptoms upon admission to the center in years 2011–2018. **Results:** In total, 783 antibiograms were analyzed for *Klebsiella pneumoniae* (258 samples, 33.0%), *Escherichia coli* (212, 27.0%), *Enterococcus faecalis* (128, 24.0%), and *Enterococcus faecium* (125, 16.0%). The decrease in susceptibility of *E. coli* to amoxicillin/clavulanic acid (62.9% vs. 40.0%) and ciprofloxacin (100% to 40.0%) was observed. Susceptibility to gentamicin increased from 33.3% to 92.9% in *E. faecium*. Susceptibility to tigecycline remained 100% through all years in case of *E. faecalis* and *E. faecium*. Male gender was a RF for resistance to amoxicillin/clavulanic acid (*p* = 0.008), ciprofloxacin (*p* = 0.0003), trimethoprim/sulfamethoxazole (*p* = 0.00009), ceftriaxone (*p* = 0.0001), and cefuroxime axetil (*p* = 0.00038) in *K. pneumoniae* and against gentamicin in *E. faecalis* (*p* = 0.015). Higher resistance to ampicillin in *E. faecalis* (*p* = 0.012) and to ciprofloxacin (*p* = 0.0003), trimethoprim/sulfamethoxazole (*p* = 0.007), piperacillin/tazobactam (*p* = 0.003), ceftriaxone (*p* = 0.001), and cefuroxime axetil (*p* = 0.013) in *K. pneumoniae* was observed in higher age groups of patients. Diabetes as a cause of kidney insufficiency (*p* = 0.026) and kidney-pancreas transplantation (*p* = 0.014) was RF for resistance to ceftriaxone in *K. pneumoniae*. **Conclusions:** AMR in uropathogens from KTX recipients fluctuated. There were identifiable RFs for resistance in the examined bacteria–antibiotic combinations. We recommend continuous mapping of site-specific microorganisms as etiology and susceptibility may vary between institutions and over time.

## 1. Introduction

Antimicrobial resistance (AMR) is a global problem in various healthcare institutions [1,2], yet organ recipients are especially at risk when confronted with resistant bacteria [3,4]. Transplant patients are a vulnerable group due to immunosuppressive medicines they receive to prevent organ rejection. Together with altered anatomy of the operated site, present catheters, or comorbidities it makes, such patients that are more susceptible to infections often must be treated with wide-spectrum antibiotics [3,5,6]. The most common solid organ transplanted is kidney, and bacterial urinary tract infections (UTIs) are among the most frequent causes for admission to the hospital among renal graft recipients [7,8]. Prophylactic and diagnostic strategies are available to physicians treating UTIs in patients after kidney transplantation (KTX) [9,10,11]. Clinicians have a range of recommendations concerning prevention, diagnostic, and treatment protocols concerning UTI on the national (Poland) [12] and European [13] level. However, what recommendations cannot fully adapt to is population-specific and local etiology of uropathogens, which may differ significantly between transplant centers. Gołębiewska et al. (2011) mapped out that predominant bacteria causing UTI in patients after KTX at the Medical University of Gdansk, Poland were *Enterococcus faecium* and *Escherichia coli* [14]. Korth and co-workers (2017) identified dominant etiology of *E. coli*, *Klebsiella spp.,* and *Pseudomonas aeruginosa* at the University Hospital Essen, Germany [15]. Pellé et al. (2007) found that in Tenon Hospital in Paris, France the most frequent uropathogens in the studied population were *E. coli*, *P. aeruginosa*, and *Enterococcus spp.* [16]. Local and population-specific antibiograms like those presented in this article can provide more precise and customized support concerning the empirical management of infections in particular groups such as transplant patients [17]. It can further contribute to antimicrobial stewardship as recommended by the World Health Organization (WHO, Global Action Plan on Antimicrobial Resistance, 2015) [18,19]. In the era of wide-spread antimicrobial resistance, prudent use of antimicrobials is of unprecedented importance.

There are 22 transplant centers in Poland where kidney transplantation procedures are performed or where patients are referred to post-transplantation in case of infections [20]. With around 1000 kidney transplantations per year in Poland (993 in 2019, POLTRANSPLANT [21]), UTIs treatment in this growing patient population is a daily challenge for all clinicians involved.

The purpose of this study was to (1) present susceptibility to selected antibiotics in four most common pathogens responsible for UTIs in KTX recipients in one transplant center in Poland as well as (2) to identify potential patient- and transplant-specific risk factors for AMR in the studied group. Findings of this study such as the frequency of pathogens, their antibiotic susceptibility rates as well as risk factors analyzed can provide additional guidance in empiric treatment of KTX recipients with UTIs in our center, especially when no clinical history of infections is available. It often occurs that when no previous antibiograms are available for a particular patient, it is necessary to use local antibiograms (e.g., for the hospital, if there are any) or general recommendations (e.g., for Poland) that might not always reflect the specificity of the KTX population. Initiating with empiric therapy in such cases without reaching out to wide-spectrum antibiotics can be informed by antibiograms of maximum group-focus, as provided in the article.

## 2. Results

We analyzed antibiograms for 783 samples of urine positive for bacterial growth of 10^5^ colony-forming units (CFU)/mL from adult patients after KTX presenting with signs or symptoms of UTI upon admission. UTI in all cases was the main diagnosis and cause for referral to the hospital. Four most common bacterial etiologies identified were: *Klebsiella pneumoniae (K. pneumoniae)* (258 samples, 33.0%), *Escherichia coli (E. coli)* (212 samples, 27.0%), *Enterococcus faecalis (E. faecalis)* (128 samples, 24.0%), and *Enterococcus faecium (E. faecium)* (125 samples, 16.0%). Information on the isolates are presented in Table 1. Other bacteria were excluded from our study as they were much less common.

### 2.1. Susceptibility to Selected Antibiotics in K. pneumoniae

For *K. pneumoniae*, there were significantly more resistant strains in males than females in case of amoxicillin/clavulanic acid (*p* = 0.008), ciprofloxacin (*p* = 0.0003), trimethoprim/sulfamethoxazole (*p* = 0.00009), ceftriaxone (*p* = 0.0001), and cefuroxime axetil (*p* = 0.00038). In terms of age of patients, there was a higher number of resistant strains in patients from older age groups (50 years or more) for ciprofloxacin (*p* = 0.0003), trimethoprim/sulfamethoxazole (*p* = 0.007), piperacillin/tazobactam (*p* = 0.003), ceftriaxone (*p* = 0.001), and cefuroxime axetil (*p* = 0.013). In the analysis for the number of years post-tx when the analyzed UTI occurred, more resistant strains were noticed in older graft groups (11 years post-tx or more) in case of amoxicillin/clavulanic acid (*p* = 0.037). For trimethoprim/sulfamethoxazole, recipients of kidneys with UTI up to 10 years post-tx had higher prevalence of resistance that was then balanced by with susceptible strains in older groups. For the type of transplantation, in case of UTIs in kidney-pancreas transplant recipients, more strains were resistant to ceftriaxone (*p* = 0.014) in comparison with other antibiotics or co-transplanted organs. Considering underlying kidney disease analyzed only for *K. pneumoniae* samples in our study, pre-existing diabetes was a risk factor for resistance to ceftriaxone compared to other end-stage kidney diseases (Table 2) that led to KTX (*p* = 0.026).

**Figure 1:**
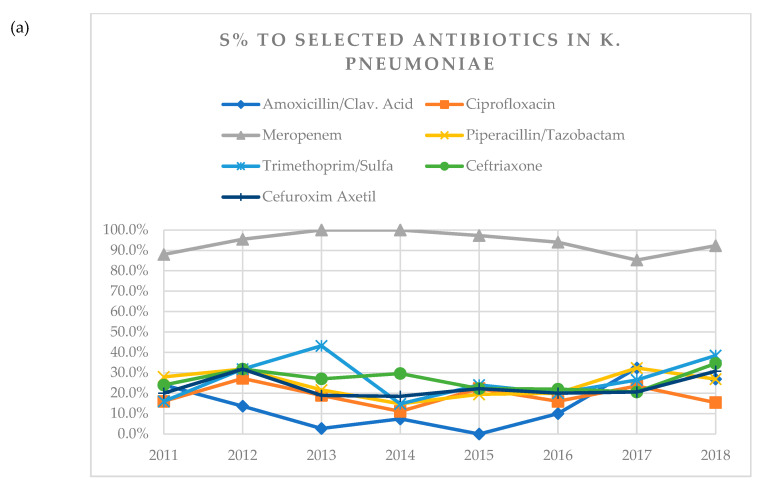

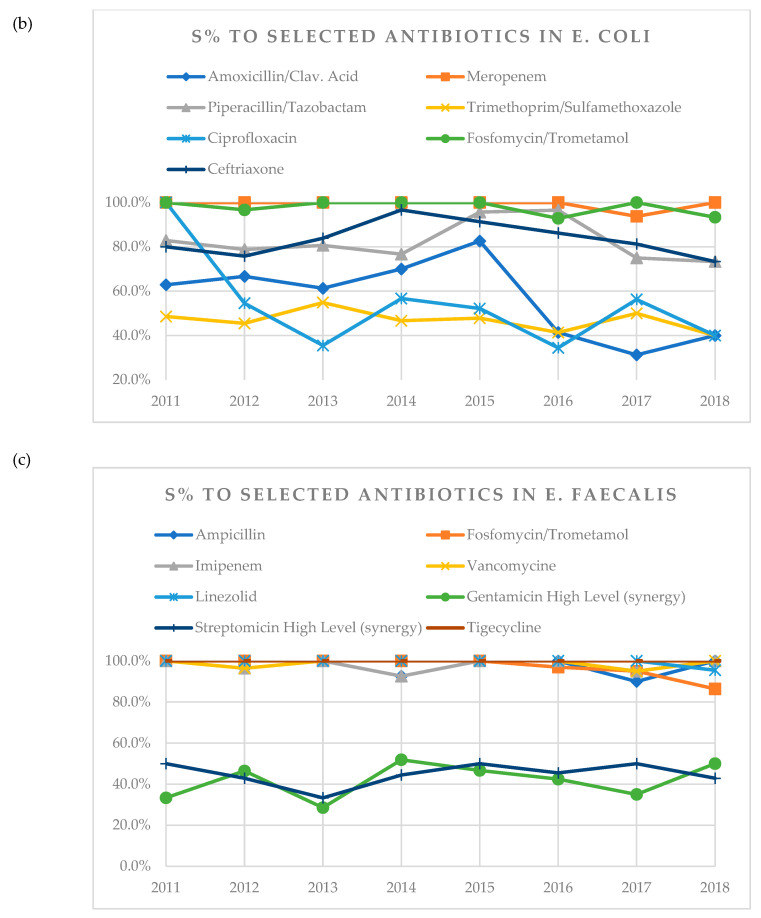

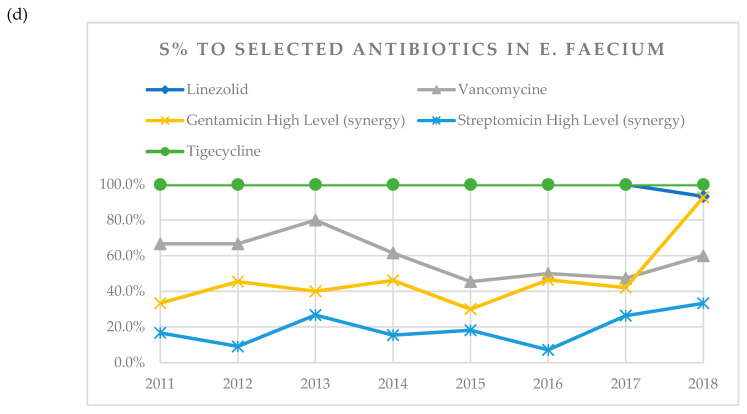
(**a**) A visual representation of changes in susceptibility (S%) of *K. pneumoniae* to tested antibiotics over years 2011–2018. (**b**) Visual representation of changes in susceptibility (S%) of *E. coli* to tested antibiotics over years 2011–2018. (**c**) Visual representation of changes in susceptibility (S%) of *E. faecalis* to tested antibiotics over years 2011–2018. (**d**) Visual representation of changes in susceptibility (S%) of *E. faecium* to tested antibiotics over years 2011–2018.

Susceptibility of *K. pneumoniae* to amoxicillin/clavulanic acid decreased over time from 24.0% in 2011 to 0.0% in 2015 and went up again to 26.9% in 2018 (Table 3, Figure 1a). No other statistically significant relationships were found between resistance and susceptibility rates of *K. pneumoniae* to other antibiotics examined and gender, age of patient, years post-tx, and number of KTX if KTX was performed together with other organs, a type of donor, or underlying kidney disease.

### 2.2. Susceptibility to Selected Antibiotics in E. coli

In case of *E. coli*, susceptibility to amoxicillin/clavulanic acid decreased over time from 62.9% in 2011 to 40.0% in 2018. Susceptibility to ciprofloxacin decreased from 100% in 2011 to 40.0% in 2018 (Table 4, Figure 1b). In the analysis for the number of years post-tx when the antibiograms were obtained, more resistant strains were noticed in older graft groups (11 or more years post-tx) in case of meropenem (*p* = 0.014) and piperacillin/tazobactam (*p* = 0.006), whereas for trimethoprim/sulfamethoxazole (*p* = 0.016) and ciprofloxacin (*p* = 0.02), resistance decreased with the number of years post-tx.

No other statistically significant relationships were found between resistance and susceptibility rates of *E. coli* to other antibiotics examined and gender, age of patient, years post-tx, and number of KTX if KTX was performed together with other organs or a type of donor.

### 2.3. Susceptibility to Selected Antibiotics in E. faecalis

In case of *E. faecalis*, susceptibility to tigecycline remained 100% throughout the whole observation period (Table 5, Figure 1c). In terms of gender, there were more resistant strains in males than females for gentamicin (*p* = 0.015). In the analysis for the age of patients, there was an increasing number of resistant strains to ampicillin for patients younger than 29 years and over 50 years (*p* = 0.012). In the analysis for the number of years post-tx, decreasing number of resistant strains was noticed with increasing number of years post-tx in case of streptomycin (*p* = 0.019). There was one urine sample where antibiogram showed resistance to linezolid, which was obtained in 2018 in a patient after 3rd KTX.

No other statistically significant relationships were found between resistance and susceptibility rates of *E. faecalis* to other antibiotics examined and gender, age of patient, years post-tx, number of KTX, if KTX was performed together with other organs or a type of donor.

### 2.4. Susceptibility to Selected Antibiotics in E. faecium

In case of *E. faecium*, susceptibility to tigecycline remained 100% throughout the whole observation period (Table 6, Figure 1d). Susceptibility to gentamicin increased over time from 33.3% in 2011 to 92.9% in 2018. All tested isolates of *E. faecium* were resistant to ampicillin and imipenem and were not included in Table 6.

No other statistically significant relationships were found between resistance and susceptibility rates of *E. faecium* to other antibiotics examined and gender, age of patient, years post-tx, and number of KTX if KTX was performed together with other organs or a type of donor.

## 3. Discussion

According to the authors’ knowledge, this is the most comprehensive study presenting analysis of different cumulative antibiograms of pathogens causing urinary tract infections specifically in kidney transplant recipients in Poland. This tool provides information on what changes in AMR occurred over 8 years of observation in our center and which patient groups among KTX recipients were characterized by higher rates of resistance in uropathogens causing infections.

### 3.1. The Importance of Presenting Population-Specific Susceptibility Reports

Susceptibility of bacteria causing infections in solid-organ transplant (SOT) recipients may be lower than the institution-specific recommendations foresee. Rosa et al. (2016) [17] presented that susceptibility of *E. coli* causing UTIs in SOT recipients against trimethoprim/sulfamethoxazole, levofloxacin, and ceftriaxone was significantly lower than the institution-wide susceptibility. In case of trimethoprim/sulfamethoxazole, the susceptibility for *E. coli* from urine was below 25% in SOT compared to over 50% according to the hospital antibiograms. Differences between transplant recipients and non-KTX population in Poland can also be pronounced. Stefaniuk et al. (2016) [22] studied urinary bacterial isolates collected from adult patients over two months of 2013 from 41 centers in all regions of Poland. Out of 156 isolates of *E. coli* obtained from patients with complicated UTI, 85.9% were susceptible to amoxicillin/clavulanic acid (61.3% in our sample in 2013), 88.5% to piperacillin/tazobactam (80.6%), 62.8% to trimethoprim/sulfamethoxazole (54.8%), 58.3% to ciprofloxacin (35.5%), and 62.2% to fosfomycin (100%). For meropenem, all samples were susceptible in both studies (100%). In conclusion, for the majority of antibiotics, except for fosfomycin and meropenem, the susceptibility rates were lower in our isolates, supporting the specificity of KTX recipients when treating UTI infections compared to the general population. Overall, site- and population-targeted antibiograms allow for more precise adaptation of antibiotic therapy considering AMR trends in specific departments.

Bacterial infections in SOT recipients often require scaling-up antibiotic treatment. Clinical decision-making may also be more demanding in case of non-SOT patients with sepsis [23]. We compared susceptibility rates in our samples with annual surveillance data from the European Centre for Disease Prevention and Control (ECDC) on invasive isolates (e.g., blood infections) collected from Poland for years 2015–2018 [24]. In case of *K. pneumoniae*, percent of resistant isolates was higher in our sample in each year for fluoroquinolones (78% vs. 64% in 2015, 85% vs. 68% in 2018). Resistance of *K. pneumoniae* to carbapenems and third-generation cephalosporins in our sample was higher in years 2015–2017 but similar to the national data in 2018 (8% and 65%, respectively). In case of *E. coli*, percent of resistant isolates in our sample was much higher in each year for fluoroquinolones (48% vs. 28% in 2015, 66% vs. 33% in 2016, 44% vs. 36% in 2017, and 60% vs. 35% in 2018). For ciprofloxacin, the increase in resistance we observed was echoed in the ECDC trends. Resistance to carbapenems was only higher in our sample in 2016 when one isolate was detected with no susceptibility to meropenem (6% vs. 0%), otherwise, it was reported 0% in other years in both analyses. In case of third-generation cephalosporins, interestingly, in 2016, resistance to ceftriaxone in our sample was lower compared to the ECDC data (9% vs. 12%) but gradually increased and showed significantly higher values in 2018 (27% vs. 17.6%). Resistance in *E. faecalis* to gentamicin and in *E. faecium* to vancomycin in our sample was higher for all years compared to data provided by the ECDC. Bearing in mind that our sample size was limited, in most cases, resistance of uropathogens in patients after KTX in our clinic was higher than resistance in invasive isolates from the hospitalized population in Poland in years 2015–2018.

### 3.2. Differences and Trends in Etiology of UTIs in KTX Recipients between Transplant Centers

The choice of microorganisms in our study was based on microbiological statistics of the hospital and ordered by decreasing number of isolates per species: *Klebsiella pneumoniae* (1), *Escherichia coli* (2), *Enterococcus faecalis* (3), and *Enterococcus faecium* (4). Uropathogens statistics presented by Gozdowska et al. (2016) [25] analyzed for the same center between October 2013 and October 2014 showed different results. The authors studied epidemiology of UTIs in KTX recipients and identified that the most common pathogens in our center in that period were *E. coli* (1), *K. pneumoniae* (2), and *Enterococcus spp.* (3). In our study, *K. pneumoniae* was the most common single pathogen identified, exceeding *E. coli* (258 vs. 212 urine samples) when the study comprised longer period (2013–2014 vs. 2011–2018). Other transplant centers may have a different composition of etiologies of UTIs in KTX recipients [14,15,16], thus requiring different treatment approaches.

As signaled earlier, we noticed in our study that the number of isolates of a given microorganism was changing along the observed period. The number of UTIs caused by *E. coli* was decreasing, whereas *K. pneumoniae* showed an increase. Changes in etiology over time were also noticed by Origüen et al. (2016) [26] who compared UTI etiologies in two groups of KTX recipients—those who received a transplant between 2002–2004 (A) and between 2011–2013 (B). Both groups were followed up for 2–3 years post-tx. In that study, the number of *K. pneumoniae* isolates went from 9.5% in group A (earlier) to 15.6% in group B (later). *E. coli* remained the single most common pathogen in both cohorts but its contribution to the overall epidemiology decreased (A—59.5% vs. B—46.5%). Individual changes in compositions of uropathogens in KTX recipients depending on the period from transplantation were studied [16,27] but data on such population shifts in etiology in this group of patients is scarce. The latter could be due to introduction of newer immunosuppressive regimens as suggested by Alangaden and co-workers (2006) [28] or by aging of the population, including transplant patients [29], which, for instance, impacts the human microbiome [30]. More studies are needed to define the dynamics of this process and whether it can be observed in other transplant centers.

Pathogens responsible for UTI in KTX can differ within the same center over diverse periods and between transplant departments of various hospitals. This further underlines the importance of collecting data on epidemiology of infections and susceptibility of microorganisms specific to the site and population examined.

### 3.3. Comparison of Susceptibility Patterns with Other Studies

Based on our data, in case of *K. pneumoniae* and *E. coli*, significant decrease was noticed in susceptibility to ciprofloxacin over the observed period reaching 15.4% and 40.0%, respectively, in 2018. Based on hospital antibiograms made in that year for all patients in all departments, susceptibility to ciprofloxacin in our institution was 69% for *K. pneumoniae* and 41% for *E. coli*. This shows that *K. pneumoniae* strains obtained from urine samples of KTX patients in our department are more resistant to fluoroquinolones compared to samples from other departments of the same hospital (susceptibility: 15.4% vs. 69%).

In a study by Łazińska et al. (2005) [31], describing the population of KTX recipients in an outpatient clinic of the same hospital in years 1995–2001, there were 22 antibiograms of *E. coli* presented with calculated average susceptibility of 90.5% to ciprofloxacin and 100% to ceftriaxone. In our study, analyzing urine samples from a similar population of patients but 10–23 years later, average susceptibility decreased to 55.7% for ciprofloxacin and 84% for ceftriaxone. It implies that over 20 years the resistance to selected antibiotics in comparable local groups of KTX recipients progressed (from 90.5% to 55.7% for ciprofloxacin and from 100% to 84% for ceftriaxone). However, low number of isolates from the latter study (22 samples) calls for prudence in making firm conclusions based on comparison with our data.

Other authors also noticed significant increase in resistance to antibiotics in urinary pathogens in KTX recipients over time. Korth et al. (2017) [15] described decline in susceptibility in *K. pneumoniae* to ciprofloxacin by 15% (*p* = 0.01), ceftazidime by 17% (*p* = 0.004), and trimethoprim/sulfamethoxazole by 19% (*p* = 0.02), based on urine samples obtained in years 2009–2012 in Essen, Germany. Azap et al. (2013) [32] observed increase of resistance to ciprofloxacin in *E. coli* by 29% based on urine samples obtained from KTX patients in Ankara, Turkey between 2003 and 2012, which resonates with findings in our sample where susceptibility to ciprofloxacin decreased from 100% in 2011 to 40.0% in 2018. The problem of resistance to cephalosporins and fluoroquinolones of analyzed bacteria marks an increase also in non-transplant patients, including non-hospitalized individuals presenting with common infections [33,34,35].

In our study, susceptibility to gentamicin in *E. faecium* varied between minimum 30% and maximum 46% in years 2011–2017 and increased rapidly to 93% in 2018. Gentamicin is a rarely used antibiotic in treatment of patients with decreased kidney function and KTX recipients due to its nephrotoxicity, which may explain such a favorable change in susceptibility. Nonetheless, such a dynamic rise in the final year of observation was unforeseen and would require further examination. Some authors recommend considering aminoglycosides in combination therapy with other antibiotics to treat UTI caused by multi-drug resistant (MDR) bacteria also in KTX recipients [3]. This is due to the spread of MDR organisms that increasingly often force clinicians to consider therapeutic options reserved as second- or third-line antibiotics.

### 3.4. Antimicrobial Resistance in Relation to Patient-Dependent and Transplant-Dependent Factors

#### 3.4.1. Gender of Recipients

In our study, male gender was a statistically significant risk factor for resistance against amoxicillin/clavulanic acid, ciprofloxacin, trimethoprim/sulfamethoxazole, ceftriaxone, and cefuroxime axetil in *K. pneumoniae* and against gentamicin in *E. faecalis*. This echoes findings in the general population published by Gomila et al. (2018) [35] who analyzed MDR Gram-negative bacteria causing complicated UTI of 948 patients in eastern Europe, Turkey, and Israel. According to the CANWARD Surveillance Study (2011) [36] led by Canadian Antimicrobial Resistance Alliance (CARA) [37], presenting annual Canadian AMR surveillance report, resistance of *E. coli* to nitrofurantoin and trimethoprim/sulfamethoxazole was higher in males. This could be due to higher rates of complicated UTIs in males that require longer hospitalizations and more intense antimicrobial treatment [38]. Kidney transplantation and immunosuppressive regimen cause additional risk load. This all means that male KTX recipients could be more prone to infections with resistant microorganisms as they would require scaling up antimicrobial regimen with potentially prolonged stays in transplant centers.

#### 3.4.2. Age of Recipients

Increasing age of patients in our study was a risk factor for resistance to ampicillin in *E. faecalis* (for patients over 50 years) and to ciprofloxacin, trimethoprim/sulfamethoxazole, piperacillin/tazobactam, ceftriaxone, and cefuroxime axetil in *K. pneumoniae*. Higher rates of resistance among urinary pathogens in older patients were also reported in CANWARD Surveillance Study [36] in *E. coli* in relation to amoxicillin/clavulanic acid, ciprofloxacin, nitrofurantoin, and trimethoprim/sulfamethoxazole. This was further confirmed in a large multinational study by Ben-Ami et al. (2009) [38], where production of extended-spectrum β-lactamase producing (ESBL) *Enterobacteriaceae* responsible for UTIs in non-transplant patients treated in ambulatory care showed higher resistance rates in the elderly (65+ years). Residents of tertiary-care hospitals, most often comprising senior patients, are also subject to infections with bacteria showing increasing resistance as studied by Muntean et al. (2018) [39]. Authors underline that present comorbidities add up to the risk of resistance in pathogens causing UTI, which often concerns transplant patients and ensues with age.

#### 3.4.3. Underlying Kidney Disease of Recipients

In *K. pneumoniae*, pre-existing diabetes was a risk factor for resistance to ceftriaxone (*p* = 0.026). Pouch et al. (2015) [40], who examined 1852 KTX recipients, concluded that in patients with diabetes mellitus *K. pneumoniae* strains causing bacteriuria were more frequently resistant to carbapenems than in patients without diabetes. Pre- or post-transplant diabetes was increasing the odds for resistance to quinolones and production of ESBL in a study on 555 urine samples by Delmas-Frenette et al. (2017) [41]. Since episodes of UTI in patients with diabetes are more common and severe, it often poses the need to intensify antibiotic treatment, which translates into higher risk of resistant strains [42]. Moreover, type 1 diabetes patients qualified for transplantation are often considered for simultaneous kidney-pancreas transplantation, which is an independent risk factor for multi-drug resistance in bacteria causing post-tx UTI [43]. In literature, we find that, in general, transplantation of “1+ organs” is a risk factor for AMR [40]. In our study, *K. pneumoniae* showed statistically higher resistance to ceftriaxone (*p* = 0.014) in kidney-pancreas recipients compared to UTIs in recipients of other co-transplanted organs or kidney alone. This means that multi-organ transplant recipients, including kidney-pancreas recipients, should be under careful monitoring considering infections especially with drug-resistant pathogens.

### 3.5. Limitations

This study has certain limitations. One limitation is the univariate analysis used as a main statistical method, therefore the error in causative relationships described cannot be excluded. However, these methods were consulted and justified by sample sizes of the analyzed groups and used intentionally. Furthermore, the number of samples obtained was lower in some cases than compared to other studies and we could not always follow the “30 isolates per specimen per year” rule as recommended by the Clinical and Laboratory Standards Institute (CLSI). KTX recipients are a narrow group of patients and samples came from a single transplant center. Moreover, many urine samples taken correctly are negative even when UTI is strongly suspected based on clinical findings. It also sporadically occurred that the number of samples tested for each antibiotic varied in a given year. For example, for *E. coli* in case of fosfomycin (FOF) in 2016 there are 28 samples and for other antibiotics in that year there are 29 samples. Such missing data were caused either by the lack of reagent in a given day, omission by a lab worker when uploading antibiograms into the system, or by the fact that the antibiotic was not regularly tested in a given strain in that year. Our data could be followed up in the years to come and combined with information from other transplant departments to create more thorough susceptibility mapping.

## 4. Materials and Methods

### 4.1. Qualification of Urine Samples

Urine samples and antibiograms were obtained from adult patients hospitalized in the Department of Transplantation Medicine, Nephrology, Internal Diseases of the Medical University of Warsaw in Warsaw, Poland from 1 January, 2011 till 31 December, 2018. Upon admission to the center, all patients presented with signs and/or symptoms of UTI (e.g., body temperature >37.0 °C, pain in the graft area, change in urinary urgency, frequency, look, or smell of urine) or abnormalities in laboratory results performed ambulatorily (leukocyturia, elevated C-reactive protein, leukocytosis or elevated serum creatinine). UTI was considered primary diagnosis with which patients were admitted to our center.

Cultures were grown by the microbiology laboratory of the same hospital as our transplant center using routine methods as described further in this section. Construction and analysis of cumulative antibiograms were made in accordance with CLSI guidelines based on a document M39-A entitled “Analysis and Presentation of Cumulative Antimicrobial Susceptibility Test Data” [44] applying the following rules:

- In case a patient was hospitalized due to UTI with positive urine culture twice or more times within an administrative year, only the first antibiogram was included in the analysis and other samples from the same patient in that year were excluded (duplicates). In total, there were 783 samples we obtained from the laboratory in administrative years 2011–2018. After excluding duplicates, we were left with 723 samples as presented in the final analysis.
- Only final and verified results were used.
- No surveillance isolates were included.
- There were no temporal outbreaks of analyzed bacteria that might have affected the result.

Due to high specificity of the examined group of patients, the rule of at least 30 isolates per species (CLSI) was not possible to follow in all cases. For the purpose of our analysis, we decided that “Intermediate” and “Resistant” isolates would be grouped.

Detailed results are presented in Section 2.1, Section 2.2, Section 2.3 and Section 2.4 (Table 3, Table 4, Table 5 and Table 6) in the % of susceptible isolates (%S). Statistically significant results for the analysis of resistance and susceptibility patterns for gender, age of patient, years from KTX procedure (post-tx), number of KTX (how many grafts at the time of UTI), if KTX was performed together with other organs or a type of donor (living vs. deceased, related vs. unrelated) for all bacteria were presented in Section 2.1, Section 2.2, Section 2.3 and Section 2.4. For *K. pneumoniae* (Table 3), the underlying cause of kidney insufficiency was additionally analyzed as an independent variable since it comprised the biggest set of data from antibiograms. In the studied *Enterococci*, fosfomycin/trometamol has registration for use in cases of *E. faecalis* only thus was not included in the analysis of *E. faecium* isolates (Table 6). Results not statistically significant were not included in descriptions but all data is available in Supplementary Materials S1–S4.

### 4.2. Microbiology and Laboratory Methods

Clean catch mid-stream urine samples taken upon admission were inoculated with a sterile calibrated loop (0.001 mL) on MacConkey agar, Sabouraud agar, and chromogenic CPS 3 plates (bioMérieux, France). Positive samples showed bacterial concentrations of at least 10^5^ CFU/mL were subjected for further analysis—identification and susceptibility testing.

Microbial identification had been performed based on biochemical test with the use of the automated system VITEK^®^ 2 (bioMérieux) and since 2013 by mass spectrometry with the use of matrix-assisted laser desorption/ionization–time-of-flight technology (MALDI-TOF MS), an automated mass spectrometry microbial identification system VITEK^®^ MS (bioMérieux). Reference strains had been used according to the European Committee on Antimicrobial Susceptibility Testing (EUCAST) guidelines.

Antibiotic susceptibility tests were conducted according to the Polish Reference Center for Antimicrobial Susceptibility and the EUCAST guidelines. Tests were performed on the automated system—VITEK^®^ 2 (bioMérieux). Breakpoints used for the identification of susceptibility in bacteria are provided in Table 7. Intermediate susceptibility was interpreted as the values between the S and R breakpoints. If the S and R breakpoints had the same value there was no intermediate category. Susceptibility testing to fosfomycin/trometamol had been performed according to the CLSI guidelines by Kirby-Bauer (disk-diffusion) method.

The selection of antibiotics presented in the study was based on the most common empiric treatment choices upon admission to the transplant center and practical suggestions of the Department of Medical Microbiology of the same hospital. We discussed with physicians and microbiologists which cumulative antibiograms would prove most useful in their work and which would they most likely consult before choosing an antibiotic for a hospitalized KTX patient with symptoms of UTI without previous cultures available.

### 4.3. Statistical Analysis

The data were analyzed with SPSS version 25 (IBM, Armonk, NY, USA). Quantitative variables were presented as absolute values and percent values calculated for each bacteria strain (*n*) and for total number of all samples (N). Susceptibility to antibiotics in analyzed bacteria was presented as percent with 95% confidence intervals (95% CI). For the analysis of risk factors in Section 2.1, Section 2.2, Section 2.3 and Section 2.4, *p*-value was calculated using the independent samples of Pearson’s chi-squared test as well. The use of other statistical methods, in this case, was limited by the size of samples. Statistical significance was based on the criterion *p* < 0.05. Data and calculations are provided in the Supplementary Materials S1–S4, including curve estimation for all antibiotics presented (Spreadsheets S1b–S4b)

### 4.4. Ethical Statement

The study was conducted in accordance with the Declaration of Helsinki, and the protocol was approved by the Ethics Committee of the Medical University of Warsaw in Warsaw, Poland (AKBE/62/2019, 18 February 2019).

## 5. Conclusions

Antimicrobial resistance in uropathogens from kidney transplant recipients has variable dynamics depending on the bacteria and antibiotics analyzed. Male gender, increasing age of the recipient, kidney-pancreas transplantation, and diabetes as underlying kidney disease were risk factors for AMR regarding certain antibiotics in *K. pneumoniae, E. coli, and Enterococci* in our sample. Epidemiology of UTIs in KTX recipients was shifting with observed increasing number of *K. pneumoniae* isolates over time. Susceptibility of bacteria among kidney transplant recipients in 2018 was lower than in the institution-wide population. To complement the observed trends for AMR in pathogens causing UTIs in KTX recipients, we suggest a continuous record of antimicrobial susceptibility in the following years in order to keep clinicians better informed about local etiology and optimal targeted therapeutic choices.

## Figures and Tables

**Table 1:** Information on all isolates grouped by patient characteristics and types of kidney transplants received.

Category		*K. pneumoniae*		*E. coli*		*E. faecalis*		*E. faecium*		Total
		*n* (% of *n*)	% of Samples N	*n* (% of *n*)	% of Samples N	*n* (% of *n*)	% of Samples N	*n* (% of *n*)	% of Samples N	N
		258 (100%)	33.0%	212 (100%)	27.0%	188 (100%)	24.0%	125 (100%)	16.0%	783
**Gender**	Males	130 (50.4%)	40.4%	60 (28.3%)	18.6%	86 (45.7%)	26.7%	46 (36.8%)	14.3%	322
	Females	128 (49.6%)	27.8%	152 (71.7%)	33.0%	102 (54.3%)	22.1%	79 (63.2%)	17.1%	461
**Age of a patient [y]**	<29	20 (7.7%)	36.4%	13 (6.1%)	23.6%	10 (5.3%)	18.2%	12 (9.6%)	21.8%	55
	30–39	40 (15.5%)	29.9%	31 (14.6%)	23.1%	43 (22.9%)	32.1%	20 (16.0%)	14.9%	134
	40–49	41 (15.9%)	33.6%	39 (18.4%)	32.0%	25 (13.3%)	20.5%	17 (13.6%)	13.9%	122
	50–59	67 (26.0%)	34.4%	49 (23.1%)	25.1%	48 (25.5%)	24.6%	31 (24.8%)	15.9%	195
	60–69	72 (27.9%)	34.0%	62 (29.3%)	29.2%	45 (24.0%)	21.2%	33 (26.4%)	15.6%	212
	70<	18 (7.0%)	27.7%	18 (8.5%)	27.7%	17 (9.0%)	26.2%	12 (9.6%)	18.4%	65
**Years from KTX procedure [y]**	<10	186 (72.1%)	37.9%	109 (51.4%)	22.2%	110 (58.5%)	22.4%	86 (68.8%)	17.5%	491
	11–20	54 (20.9%)	25.2%	79 (37.3%)	36.9%	56 (29.8%)	26.2%	25 (20.0%)	11.7%	214
	21<	16 (6.2%)	22.9%	22 (10.4%)	31.4%	18 (9.6%)	25.7%	14 (11.2%)	20.0%	70
	nd	2 (0.8%)	25.0%	2 (0.9%)	25.0%	4 (2.1%)	50.0%	0 (0.0%)	0.0%	8
Number of KTX*:	I KTX	218 (84.5%)	32.3%	186 (87.7%)	27.5%	163 (86.7%)	24.1%	109 (87.2%)	16.1%	676
	II KTX	33 (12.8%)	37.5%	22 (10.4%)	25.0%	19 (10.1%)	21.6%	14 (11.2%)	15.9%	88
	III KTX	5 (1.9%)	45.4%	2 (0.9%)	18.2%	2 (1.1%)	18.2%	2 (1.6%)	18.2%	11
	nd	2 (0.8%)	25.0%	2 (0.9%)	25.0%	4 (2.1%)	50.0%	0 (0.0%)	0.0%	8
**Type of TX: KTX alone or with other organs**	KTX alone	217 (84.1%)	32.4%	190 (89.6%)	28.4%	158 (84.1%)	23.6%	104 (83.2%)	15.6%	669
	KTX + Bricker	21 (8.1%)	32.8%	11 (5.2%)	17.2%	19 (10.1%)	29.7%	13 (10.4%)	20.3%	64
	KTX + heart	0 (0.0%)	0.0%	1 (0.5%)	50.0%	1 (0.5%)	50.0%	0 (0.0%	0.0%	2
	KTX + pancreas	16 (6.2%)	47.1%	8 (3.8%)	23.5%	4 (2.1%)	11.8%	6 (4.8%)	17.6%	34
	KTX + liver	2 (0.8%)	33.3%	0 (0.0%)	0.0%	2 (1.1%)	33.3%	2 (1.6%)	33.3%	6
	nd	2 (0.8%)	25.0%	2 (0.9%)	25.0%	4 (2.1%)	50.0%	0 (0.0%)	0.0%	8
**KTX donor: living or deceased**	Living	21 (8.1%)	43.8%	11 (5.2%)	22.9%	9 (4.8%)	18.8%	7 (5.6%)	14.6%	48
	Deceased	235 (91.1%)	32.2%	199 (93.9%)	27.3%	177 (94.1%)	24.3%	118 (94.4%)	16.2%	729
	nd	2 (0.8%)	33.3%	2 (0.9%)	33.3%	2 (1.1%)	33.3%	0 (0.0%)	0.0%	6
**KTX donor: related or unrelated**	Related	16 (6.2%)	47.0%	9 (4.3%)	26.5%	5 (2.6%)	14.7%	4 (3.2%)	11.8%	34
	Unrelated	240 (93.0%)	32.3%	201 (94.8%)	27.0%	181 (96.3%)	24.4%	121 (96.8%)	16.3%	743
	nd	2 (0.8%)	33.3%	2 (0.9%)	33.3%	2 (1.1%)	33.3%	0 (0.0%)	0.0%	6

KTX—kidney transplant, TX—transplantation, Number of KTX*—how many grafts at the time of urinary tract infection (one—I, two—II, three—III), N—total number of isolates, *n*—number of isolates for each bacteria, % of *n*—percent of isolates from a given category (left column) for each bacteria (add up to 100% vertically), % of samples N—percent of isolates calculated from a total number of isolates (N) for all bacteria (right column) in a given category (e.g., gender, add up to 100% horizontally), nd—no data, y—years.

**Table 2:** Causes of end-stage kidney disease in patients with urinary tract infections due to *K. pneumoniae* (258 samples).

Underlying Kidney Disease.	N of Patients
Glomerulonephritis	56
Urologic	44
Polycystic kidney disease	41
Diabetes mellitus type 1 or 2	39
Hypertension	12
Systemic disease * (e.g., vasculitis)	7
Focal segmental glomerulosclerosis	6
Interstitial nephritis	5
Sepsis	3
Unclear or mixed cause	33
Other	7
No data	5

* not classified as any of the other provided categories.

**Table 3:** Table presenting susceptibility (S%) to selected antibiotics of *Klebsiella* *pneumoniae* isolates collected from urine samples of KTX recipients over years 2011–2018.

S% to ATB in *K. pneumoniae*		AMC	CIP	MEM	TZP	TMP/SMX	CRO	CXM
2011 N = 25	S%	24.0%	16.0%	88.0%	28.0%	16.0%	24.0%	20.0%
	95% CI	11.5–43.4	6.4–34.7	70.0–95.8	14.3–47.6	6.4–34.7	11.5–43.4	8.9–39.1
2012 N = 22	S%	13.6%	27.3%	95.5%	31.8%	31.8%	31.8%	31.8%
	95% CI	4.7–33.3	13.2–48.2	78.2–99.2	16.4–52.7	16.4–52.7	16.4–52.7	16.4–52.7
2013 N = 37	S%	2.7%	18.9%	100.0%	21.6%	43.2%	27.0%	18.9%
	95% CI	0.5–13.8	9.5–34.2	90.6–100.0	11.4–37.2	28.7–59.1	15.4–43.0	9.5–34.2
2014 N = 27	S%	7.4%	11.1%	100.0%	14.8%	14.8%	29.6%	18.5%
	95% CI	2.1–23.4	3.9–28.1	87.5–100.0	5.9–32.5	5.9–32.5	15.9–48.5	8.2–36.7
2015 N = 37	S%	0.0%	21.6%	97.3%	18.9%	24.3%	21.6%	21.6%
	95% CI	0.0–9.4	11.4–37.2	86.2–99.5	9.5–34.2	13.4–40.1	11.4–37.2	11.4–37.2
2016 N = 50	S%	10.0%	16.0%	94.0%	20.0%	20.0%	22.0%	20.0%
	95% CI	4.3–21.4	8.3–28.5	83.8–97.9	11.2–33	11.2–33	12.8–35.2	11.2–33.0
2017 N = 34	S%	32.4%	23.5%	85.3%	32.4%	26.5%	20.6%	20.6%
	95% CI	19.1–49.2	12.4–40.0	69.9–93.6	19.1–49.2	14.6–63.1	10.3–36.8	10.3–36.8
2018 N = 26	S%	26.9%	15.4%	92.3%	26.9%	38.5%	34.6%	30.8%
	95% CI	13.7–46.1	6.2–33.5	75.9–97.9	13.7–46.1	22.4–57.5	19.4–53.8	16.5–50.0
Total N_total_ = 258	S% avg	13.6%	18.6%	94.2%	23.6%	26.7%	25.6%	22.1%
	95% CI	9.9–19.3	14.3–23.8	90.6–96.4	18.9–29.2	21.7–32.5	20.6–31.2	17.5–27.5

ATB—antibiotic name, N—number of all isolates analyzed for an ATB in a given year, S%—percent of susceptible isolates in a given year, S% avg—average susceptibility calculated for all years (*n*_total_/N_total_), AMC—amoxicillin/clavulanic acid, CIP—ciprofloxacin. MEM—meropenem, TZP—piperacillin/tazobactam, TMP/SMX—trimethoprim/sulfamethoxazole, CRO—ceftriaxone, CXM—cefuroxime axetil.

**Table 4:** Table presenting susceptibility (S%) to selected antibiotics of *Escherichia coli* isolates collected from urine samples of KTX recipients over years 2011–2018.

S% ATB in *E. coli*		AMC	MEM	TZP	SXT	CIP	FOF	CRO
2011 N = 35 N_FOF_ = 24	S%	62.9%	100.0%	82.9%	48.6%	100.0%	100.0%	80.0%
	95% CI	46.3–76.8	90.1–100.0	67.3–91.9	33.0–64.4	90.1–100.0	86.2–100.0	64.1–90.0
2012 N = 33 N_FOF_ = 30	S%	66.7%	100.0%	78.8%	45.5%	54.5%	96.7%	75.8%
	95% CI	49.6–80.2	89.6–100.0	62.2–89.3	29.8–62.0	38.0–70.2	72.7–95.2	59.0–87.2
2013 N = 31	S%	61.3%	100.0%	80.6%	54.8%	35.5%	100.0%	83.9%
	95% CI	43.8–76.3	89.0–100.0	63.7–90.8	37.8–70.8	21.1–53.1	89.0–100.0	67.4–92.9
2014 N = 30	S%	70.0%	100.0%	76.7%	46.7%	56.7%	100.0%	96.7%
	95% CI	52.1–83.3	88.6–100.0	59.1–88.2	30.2–63.9	39.2–72.6	88.6–100.0	83.3–99.4
2015 N = 23	S%	82.6%	100.0%	95.7%	47.8%	52.2%	100.0%	91.3%
	95% CI	62.9–93.0	85.7–100.0	79.0–99.2	29.2–67.0	33.0–70.8	85.7–100.0	73.2–97.6
2016 N = 29 N_FOF_ = 28	S%	41.4%	100.0%	96.6%	41.4%	34.5%	92.9%	86.2%
	95% CI	25.5–59.3	88.3–100.0	82.8–99.4	25.5–59.3	19.9–52.7	73.6–96.4	69.4–94.5
2017 N = 16	S%	31.3%	93.8%	75.0%	50.0%	56.3%	100.0%	81.3%
	95% CI	14.2–55.6	71.7–98.9	50.5–89.8	28.0–72.0	33.2–76.9	80.6–100.0	57.0–93.4
2018 N = 15	S%	40.0%	100.0%	73.3%	40.0%	40.0%	93.3%	73.3%
	95% CI	19.8–64.3	79.6–100.0	48.0–89.1	19.8–64.3	19.8–64.3	70.2–98.8	48.0–89.1
Total N_total_ = 212 N_total–FOF_ = 193	S% avg	59.4%	99.5%	83.0%	47.2%	55.7%	98.0%	84.0%
	95% CI	52.7–65.8	97.4–99.9	77.4–87.5	40.6–53.9	48.9–62.2	94.9–99.2	78.4–88.3

ATB—antibiotic name, N—number of all isolates analyzed for an ATB in a given year, S%—percent of susceptible isolates in a given year, S% avg—average susceptibility calculated for all years (n_total_/N_total_), AMC—amoxicillin/clavulanic acid, MEM—meropenem, TZP—piperacillin/tazobactam, CIP—ciprofloxacin, FOF—fosfomycin/trometamol, CRO—ceftriaxone.

**Table 5:** Table presenting susceptibility (S%) to selected antibiotics of *Enterococcus* *faecalis* isolates collected from urine samples of KTX recipients over years 2011–2018. Isolates from the year 2011 were aggregated with 2012 because of the low count in 2011 (6 isolates).

S% to ATB in *E. faecalis*		AMP	FOF	IPM	VAN	LZD	GEN HL (s)	STR HL (s)	TGC
2011 and 2012 N = 34	S%	97.1%	100.0%	97.1%	97.1%	100.0%	44.1%	44.1%	100.0%
	95% CI	85.1–99.5	89.9–100.0	85.1–99.5	85.1–99.5	89.9–100.0	28.9–60.5	28.9–60.5	89.9–100.0
2013 N = 21 N_IPM_ = 20	S%	100.0%	100.0%	100.0%	100.0%	100.0%	28.6%	33.3%	100.0%
	95% CI	84.5–100.0	84.5–100.0	83.9–100.0	84.5–100.0	84.5–100.0	13.8–50.0	17.2–54.6	84.5–100.0
2014 N = 27	S%	92.6%	100.0%	92.6%	100.0%	100.0%	51.9%	44.4%	100.0%
	95% CI	76.6–97.9	87.5–100.0	76.6–97.9	87.5–100.0	87.5–100.0	34.0–69.3	27.6–62.7	87.5–100.0
2015 N = 31 N_GEN_ = 30N_STR_ = 30	S%	100.0%	100.0%	100.0%	100.0%	100.0%	46.7%	50.0%	100.0%
	95% CI	89.0–100.0	89.0–100.0	89.0–100.0	89.0–100.0	89.0–100.0	30.2–63.9	33.2–66.8	89.0–100.0
2016 N = 33	S%	100.0%	97.0%	100.0%	100.0%	100.0%	42.4%	45.5%	100.0%
	95% CI	89.6–100.0	84.7–99.5	89.6–100.0	89.6–100.0	89.6–100.0	27.2–59.2	29.8–62.0	89.6–100.0
2017 N = 20	S%	90.0%	95.0%	95.0%	95.0%	100.0%	35.0%	50.0%	100.0%
	95% CI	69.9–97.2	76.4–99.1	76.4–99.1	76.4–99.1	83.9–100.0	18.1–56.7	29.9–70.1	83.9–100.0
2018 N = 22 N_STR_ = 21	S%	100.0%	86.4%	100.0%	100.0%	95.5%	50.0%	42.9%	100.0%
	95% CI	85.1–100.0	66.7–95.3	85.1–100.0	85.1–100.0	78.2–99.2	30.7–69.3	24.5–63.5	85.1–100.0
Total N_total_ = 188 N_total–IPM_ = 187 N_total–GEN_ = 187 N_total–STR_ = 186	S% avg	97.3%	97.3%	97.9%	98.9%	99.5%	43.3%	44.6%	100.0%
	95% CI	93.9–98.9	93.9–98.9	94.6–99.2	96.2–99.7	97.0–99.9	36.4–50.5	37.7–51.8	98.0–100.0

ATB—antibiotic name, N—number of all isolates analyzed for an ATB in a given year, S%—percent of susceptible isolates in a given year, S% avg—average susceptibility calculated for all years (*n*_total_/N_total_), AMP—ampicillin, FOF—fosfomycin/trometamol, IPM—imipenem, VAN—vancomycin, LZD—linezolid, GEN HL (s)—gentamicin High Level (synergy), STR HL (s)—streptomycin High Level (synergy), TGC—tigecycline.

**Table 6:** Table presenting susceptibility (S%) to selected antibiotics of *E. faecium* isolates collected from urine samples of KTX recipients over years 2011–2018.

S% to ATB in E. faecium		LZD	VAN	GEN HL (s)	STR HL (s)	TGC
2011 N = 12	S%	100.0%	66.7%	33.3%	16.7%	100.0%
	95% CI	75.8–100.0	39.1–86.2	13.8–60.9	4.7–44.8	75.8–100.0
2012 N = 12 N_GEN_ = 11 N_STR_ = 11	S%	100.0%	66.7%	45.5%	9.1%	100.0%
	95% CI	75.8–100.0	39.1–86.2	21.3–72.0	1.6–37.7	75.8–100.0
2013 N = 15	S%	100.0%	80.0%	40.0%	26.7%	100.0%
	95% CI	79.6–100.0	54.8–93.0	19.8–64.3	10.9–52.0	79.6–100.0
2014 N = 13	S%	100.0%	61.5%	46.2%	15.4%	100.0%
	95% CI	77.2–100.0	35.5–82.3	23.2–70.9	4.3–42.2	77.2–100.0
2015 N = 11 N_GEN_ = 10	S%	100.0%	45.5%	30.0%	18.2%	100.0%
	95% CI	74.1–100.0	21.3–72.0	10.8–60.3	5.1–47.7	74.1–100.0
2016 N = 28	S%	100.0%	50.0%	46.4%	7.1%	100.0%
	95% CI	87.9–100.0	32.6–67.4	29.5–64.2	2.0–22.6	87.9–100.0
2017 N = 19	S%	100.0%	47.4%	42.1%	26.3%	100.0%
	95% CI	83.2–100.0	27.3–68.3	23.1–63.7	11.8–48.8	83.2–100.0
2018 N = 15 N_GEN_ = 14	S%	93.3%	60.0%	92.9%	33.3%	100.0%
	95% CI	70.2–98.8	35.7–80.2	68.5–98.7	15.2–58.3	79.6–100.0
Total N_total_ = 125 N_total_–_GEN_ = 122 N_total–STR_ = 124	S% avg	99.2%	58.4%	47.5%	18.5%	100.0%
	95% CI	95.6–99.9	49.6–66.7	38.9–56.3	12.7–26.3	97.0–100.0

ATB—antibiotic name, N—number of all isolates analyzed for an ATB in a given year, S%—percent of susceptible isolates in a given year, S% avg—average susceptibility calculated for all years (*n*_total_/N_total_), LZD—linezolid, VAN—vancomycin, GEN HL (s)—gentamicin high level (synergy), STR HL (s)—streptomycin high level (synergy), TGC—tigecycline.

**Table 7:** Breakpoints used for the identification of susceptibility in bacteria for each antibiotic.

Species and Antimicrobial Agent	MIC Breakpoints (mg/L)	
***K. pneumoniae* and *E. coli***	**S≤**	**R>**
Amoxicillin/clavulanic acid	8	8
Piperacillin/tazobactam	8	16
Cefuroxime axetil	8	8
Ceftriaxone	1	2
Meropenem	2	8
Ciprofloxacin ^1^	0.5	1
Trimethoprim/sulfamethoxazole	2	4
***E. coli* and *E. faecalis***		
Fosfomycin/trometamol ^2^	≥16 mm	≤12 mm
***E. faecalis and E. faecium***		
Ampicillin	4	8
Imipenem	4	8
Vancomycin	4	4
Linezolid	4	4
Gentamicin HL ^3^	reported as S or R only ^4^	
Streptomycin HL ^3^	reported as S or R only ^4^	
Tigecycline	0.25	0.25

^1^ in 2017 the breakpoints for ciprofloxacin were changed to: S ≤ 0.25 mg/L and R > 0.5 mg/L, ^2^ the inhibition zones used for disk-diffusion method according to the guidelines of the Clinical and Laboratory Standards Institute (Kirby-Bauer method), ^3^ test for high-level aminoglycoside resistance (HLAR), ^4^ in line with specific guidelines of the European Committee on Antimicrobial Susceptibility Testing for HLAR.

## Supplementary Materials

The following are available online at https://www.mdpi.com/2079-6382/9/6/284/s1, Spreadsheet S1—*Klebsiella pneumoniae* – 2011–2018: all samples divided by years + line charts; Spreadsheet S2—*Escherichia coli*—2011–2018: all samples divided by years + line charts; Spreadsheet S3—*Enterococcus faecalis*—2011–2018: all samples divided by years + line charts; Spreadsheet S4—*Enterococcus faecium*—2011–2018: all samples divided by years + line charts; Spreadsheet S1b—*Klebsiella pneumoniae*—curve estimation model; Spreadsheet S2b—*Escherichia coli*—curve estimation model; Spreadsheet S3b—*Enterococcus faecalis*—curve estimation model; Spreadsheet S4b—*Enterococcus faecium*—curve estimation model
